# Complement inhibition by sutimlimab in cold agglutinin disease promotes red blood cell agglutination and may lead to circulatory complications, eryptosis and hemolysis

**DOI:** 10.3389/fimmu.2026.1906515

**Published:** 2026-09-15

**Authors:** Thilo Bartolmäs, Beate Mayer, Abdulgabar Salama

**Affiliations:** 1 Institute of Transfusion Medicine, Charité-Universitätsmedizin Berlin, corporate member of Freie Universität Berlin and Humboldt-Universität zu Berlin, Berlin, Germany; 2 Department of Gynecology, Charité-Universitätsmedizin Berlin, corporate member of Freie Universität Berlin and Humboldt-Universität zu, Berlin, Germany; 3 Haemostaseologicum Medicover, Berlin, Germany

**Keywords:** agglutination, AIHA (autoimmune haemolytic anemia), cold agglutinin, complement inhibition therapy, eryptosis, hemolysis, Raynaud disease, sutimlimab

## Abstract

**Background:**

Sutimlimab, an anti-C1s monoclonal antibody, is approved for the treatment of cold agglutinin disease (CAD). It prevents hemolysis by inhibiting the classical complement pathway. One of its off-target effects may be increasing circulatory complications that have not yet been characterized.

**Methods:**

We report two patients with CAD who developed unusual widespread skin cyanosis and exacerbated acrocyanosis during sutimlimab treatment. One of the two patients additionally experienced massive intravascular hemolysis, eryptosis and acute renal failure. Serological analyses were conducted using standard techniques, and eryptosis was quantified by flow cytometry with PE-labelled Annexin V.

**Results:**

Serological assays using plasma and serum from these two patients and four additional untreated patients (n=6) with CAD were performed. They demonstrated a significant increase in red blood cell (RBC) agglutination in the presence of sutimlimab as well as in the absence of complement activation.

**Conclusion:**

The observed increase in RBC agglutination under sutimlimab treatment is likely due to a substantial reduction of C3d deposition on RBCs. This mechanism also represents the most plausible explanation for the side effects observed in some patients with CAD receiving the drug, such as aggravation of circulatory complications, including acrocyanosis, Raynaud’s phenomenon, and less commonly, organ dysfunction. Inhibition of early complement components (C1-C3) may therefore not only prevent hemolysis in CAD but paradoxically also promote RBC agglutination, eryptosis, and hemolysis in certain cases. Pre-treatment serological testing with sutimlimab may help predict some of these adverse effects.

## Introduction

Cold agglutinin disease (CAD) is a rare and well-characterized form of autoimmune hemolytic anemia (AIHA) that has been recognized for decades (1–6). Recent studies have established that CAD is a clonal B-cell disorder with dual clinical presentation, consisting of complement-mediated hemolytic anemia and cold-induced circulatory symptoms. The latter results from the direct agglutination of red blood cells (RBCs) in acral vessels at low temperatures (7, 8). The causative cold autoantibodies (aab) activate the classical complement pathway, involving early components (C1-C4) and, in some cases, terminal complement components (C5-C9). The activation begins with C1q, which triggers the classical pathway via C1r and C1s. C1s cleaves C4 and C2, forming the C3 convertase (C4b2a), which promotes coating of RBCs with C3b (9, 10). This process leads to three possible outcomes: (1) extravascular hemolysis via phagocytosis of C3b-coated RBCs, (2) survival of RBCs after C3b is inactivated to C3d, or (3) intravascular hemolysis through the formation of the membrane attack complex (C5b-9) (11–13). In addition to these mechanisms, we recently identified eryptosis as a third pathological pathway of antibody-mediated RBC destruction in AIHAs (14). Eryptosis is triggered by IgM autoantibodies that activate complement and lead to the deposition of sublytic terminal complement complexes (C5b-C6, C5b-C7, and C5b-C8) on RBCs. This induces phosphatidylserine (PS) exposure, marking the cells for clearance (15). While eryptosis and RBC senescence (the removal of aged cells) are both forms of regulated cell death, they differ in their physiological roles and kinetics (16). Eryptosis facilitates the rapid clearance of infected or damaged RBCs within minutes, whereas senescence removes older RBCs over several days (17, 18). Both processes rely on PS exposure as an “eat me” signal for phagocytosis.

The therapeutic potential of complement inhibition in immune hemolysis was first demonstrated with the approval of eculizumab for paroxysmal nocturnal hemoglobinuria (PNH) in 2007 (19). Until recently, no approved anti-complement therapy was available for CAD (12, 20, 21). However, several novel agents targeting early complement components, such as C1s, have since been developed (22–26). Among these, the humanized monoclonal antibody sutimlimab, a C1s inhibitor, has been approved by the FDA and EMA for the treatment of CAD (27–30). Clinical trials have shown that sutimlimab can stabilize hemoglobin levels (≥1.5 g/dL increase) and reduce the need for transfusions. However, its efficacy depends on continuous biweekly administration, and treatment may be associated with side effects, including infections, acrocyanosis, Raynaud’s phenomenon and circulatory complications (31–33). The latter adverse effects remain obscure, and represent a potentially serious complication that could be overlooked or misinterpreted.

Complement-targeted therapies are not expected to improve non-complement-mediated symptoms, such as acrocyanosis. However, findings from the CADENZA study revealed that treatment with sutimlimab unexpectedly led to a worsening of these symptoms in some patients (29, 33). This paradoxical effect has led some authors to caution against the use of sutimlimab in patients with CAD and severe acrocyanosis (34). In this study, we aim to elucidate the underlying pathomechanism of these adverse effects and propose potential preventive strategies.

## Case reports

### Patient no. 1

A 74-year-old woman was referred to our center due to decompensated hemolysis associated with CAD. Serological testing revealed a markedly positive C3d- direct antiglobulin test (DAT) and the presence of IgM cold aab (titer in saline 1024 at 4 °C, 256 at 20 °C, thermal amplitude up to 39 °C). Additional testing excluded the presence of warm-reactive aab and cryoglobulines. Despite strict avoidance of cold exposure, the patient intermittently required blood transfusions, which she tolerated poorly. She also reported pronounced fatigue. Following an upper respiratory infection, her hemolysis worsened significantly, with a nadir Hb level of 5.9 g/dl (reference range 11.8–15.8), a peak lactate dehydrogenase (LDH) level of 791 U/l (reference range 135–250), and an unconjugated bilirubin level of 2.14 mg/dl (reference range 0.00–0.75). The patient declined RBC transfusion during this episode. Four months later, she received two doses of sutimlimab via a compassionate use program. Immediately after the first dose, the patient reported significant clinical improvement. However, four days after the second dose, she developed generalized malaise and a hypertensive crisis (blood pressure 213/95 mm Hg), requiring emergency medical care. The patient had a history of hypertension, which had previously been well controlled with medication. Her pre-treatment blood pressure was 130/60 mmHg.

Unexpectedly, therapy was also associated with a transient exacerbation of hemolysis and the development of renal insufficiency. Laboratory data demonstrated a marked increase in LDH, free hemoglobin, bilirubin and creatinine (**Figure 1**). Notably, serum complement components C3 and C4 increased following treatment, suggesting that complement activation was not responsible for the increased hemolysis (**Figure 2A**). In addition, treatment was associated with worsening acrocyanosis and the development of livedo reticularis (**Figure 2B**). Paradoxically, fatigue symptoms also increased, with the fatigue score decreasing from 20 to 17. This episode was not associated with any complement-amplifying condition, such as infection, other autoimmune diseases, or malignant hypertension prior to sutimlimab adminstration. Thus, the adverse events were attributable solely to the therapy with sutimlimab. The patient gradually recovered over the subsequent weeks with supportive erythropoietin therapy.

**Figure 1:**
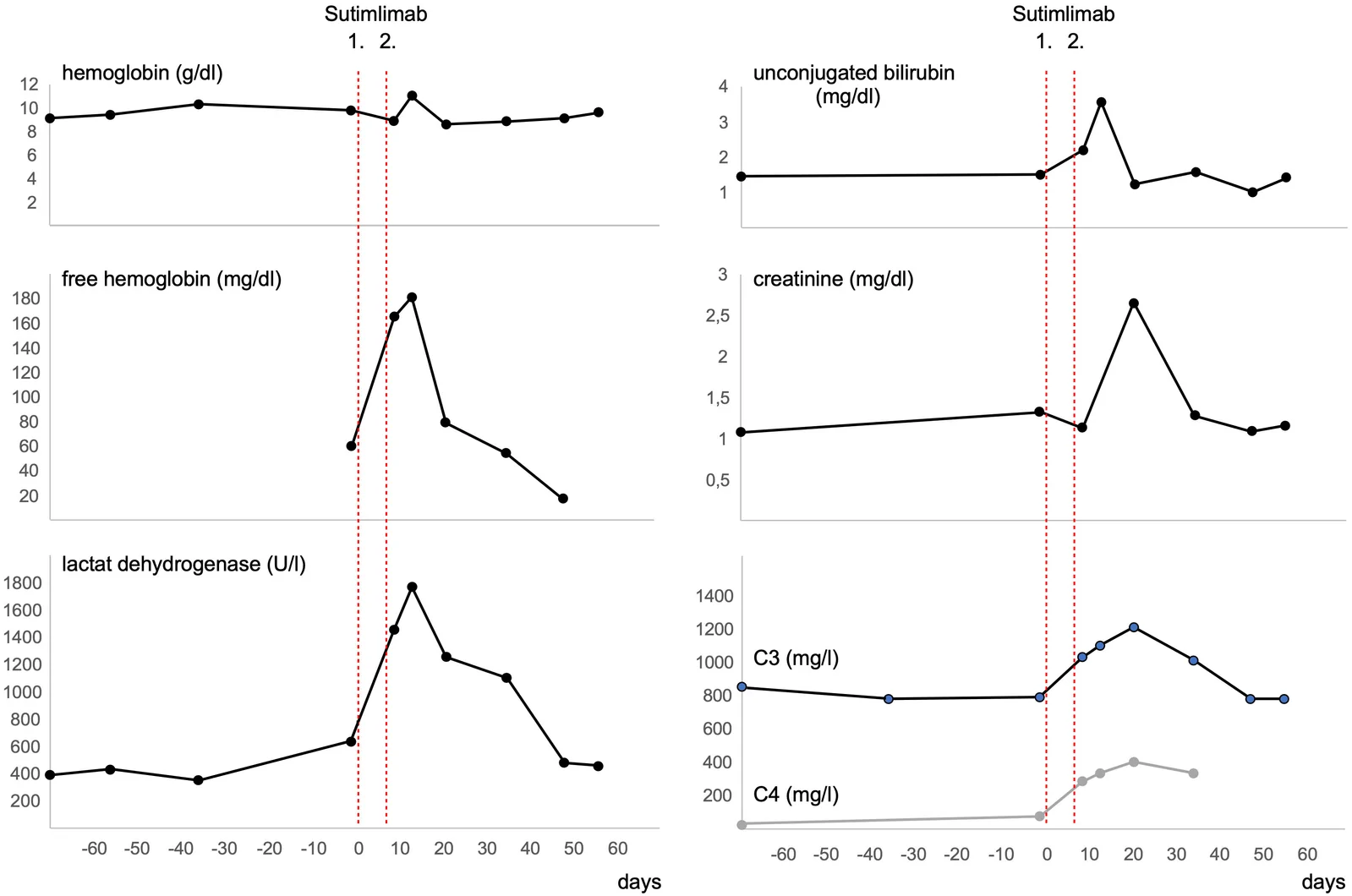
Most relevant laboratory parameters (hemoglobin, free hemoglobin, lactat dehydrogenase, unconjugated bilirubin, creatinine and complement components 3 and 4) from patient no. 1 before, during and after sutimlimab treatment.

**Figure 2:**
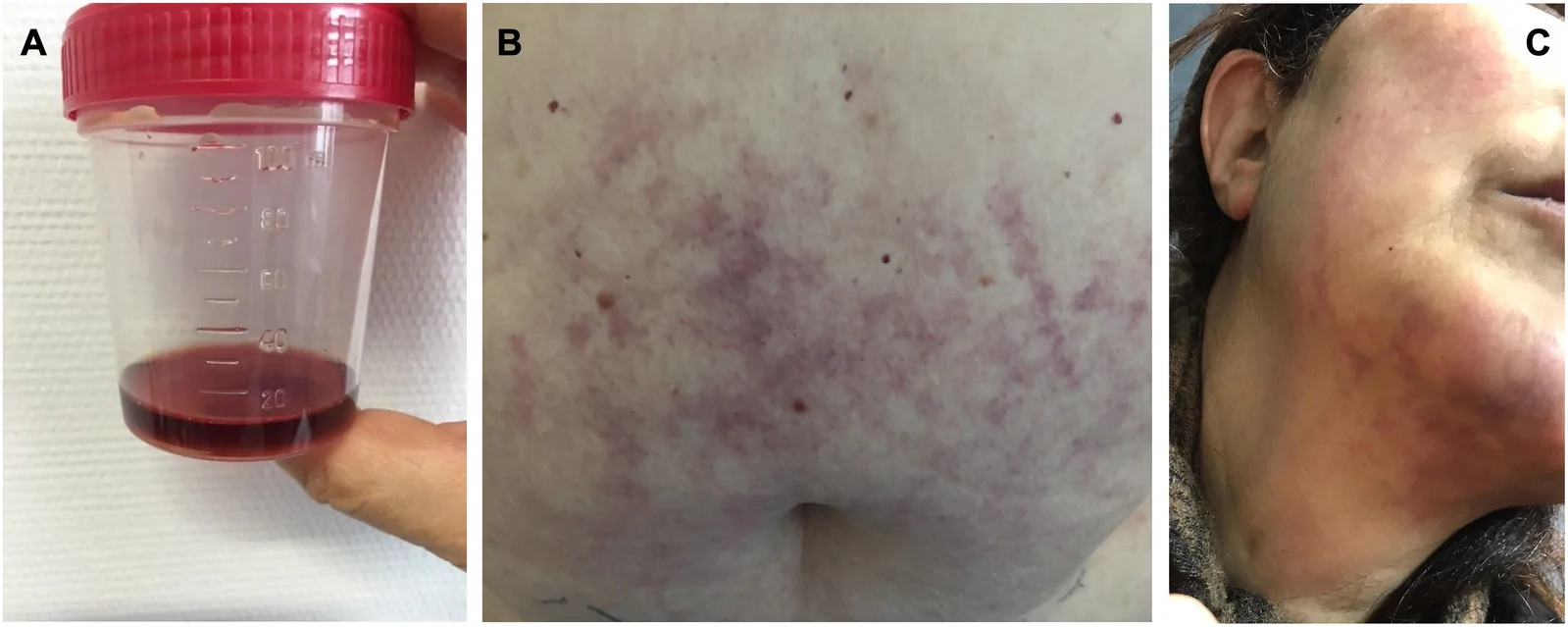
Clinical pictures of patients no. 1 and no. 2. **(A)** urine sample from patient No. 1, six days after the 2^nd^ dose of sutimlimab, with significant hemoglobinuria. **(B)** skin discoloration and livedo reticularis in patient no. 1 after the 2^nd^ dose of sutimlimab. **(C)** skin discoloration in patient no. 2 under treatment with sutimlimab.

### Patient No. 2

A 59-year-old woman has been diagnosed with CAD since late 2023. Prior to initiation of sutimlimab therapy, she experienced decompensated hemolysis, with Hb levels of 8–9 g/dl. She was referred to our medical practice due to suspected hemorrhagic events as a potential adverse effect of sutimlimab treatment. Following therapy, hemolysis improved significantly (Hb 12.7-13.2 g/dl, reticulocytes 111-113x10^3^/µl, LDH 214–257 U/l, total bilirubin 1.86-2.83 mg/dl, and haptoglobin <8 mg/dl), but she developed widespread cyanosis involving the face, neck, trunk and extremities (**Figure 2C**). Additionally, her acrocyanosis appeared to worsen during treatment period and her RBCs exhibited mild eryptosis, although hemolysis remained largely compensated. Currently, she infrequently experiences severe episodes of thoracic and abdominal pain, which appear to be related to sutimlimab therapy, as no other clinical or laboratory abnormalities were identified. She also experienced a hypertensive crisis, possibly attributable to sutimlimab treatment. In light of these adverse events, consideration should be given to whether sutimlimab therapy should be replaced with alternative agents.

## Methods

Blood samples including RBCs, serum and EDTA-plasma were collected from healthy blood donors and patients with CAD. Standard serological tests including direct antiglobulin test (DAT), indirect antiglobulin test (IAT), eluates, cold agglutinin titers and thermal amplitudes were performed as described elsewhere (35).

### Cold agglutinin titers

To allow complement activation, cold agglutinin titers were performed at room temperature (20 °C), using 50 µl of O RBCs (3% in saline) and serum or EDTA-plasma samples from patients. Patients’ serum samples were diluted in serum, and plasma samples in plasma from blood donors. After 20 min incubation at 20 °C and spinning down for 30 s, RBCs were checked for agglutination, then warm washed three times and tested for C3d-coating. The cold agglutinin titer was defined as the reciprocal value of the highest dilution with an agglutination strength of at least 1+ (36).

### Coating of RBCs with C3d

Coating of RBCs with C3d was performed by incubating 100 µl packed O RBCs with a mixture of 10 µl serum from a patient with CAD and 490 µl donor serum for 2 h at 24 °C. Subsequently, the cells were washed three times with warm saline and tested with anti-C3d.

### Testing for hemolysis, C3d-coating and eryptosis

The hemolysis assay was performed as previously described (37). Briefly, 20 µl of packed donor O RBCs were mixed with patients’ serum and diluted 1:10 in donor serum as a source of complement. The mixture was incubated for 30 minutes at 26 °C, followed by 180 minutes at 37 °C. Free hemoglobin (fHb) in the supernatant was measured photometrically with the cyanmethemoglobin method. Subsequently, the packed RBCs were re-incubated with AB donor plasma for 48 hours at 4 °C. After three warm washing steps, the RBCs were assessed for eryptosis and C3d-coating.

### *In vitro* inhibition of complement by sutimlimab

Patients’ serum samples were diluted 1:10 in fresh serum samples from blood donors, and sutimlimab (Sanofi-Aventis Deutschland GmbH, Frankfurt/Main, Germany) was added to a final concentration of 5mg/ml (1:10). After incubation for 5 min at 20 °C, 20 µl packed RBCs were added to the mixture and then checked for agglutination, hemolysis, eryptosis and C3d coating.

### Eryptosis

Measurements of RBCs for eryptosis were performed as has been described before (14). Briefly, 5 µl annexin-PE were mixed with 45 µl annexin buffer and 5 µl of 1% RBCs suspension was added. After 15 min of incubation, 450 µl annexin buffer were added and RBCs were analyzed by flow cytometry using a MacsQuant analyzer (Miltenyi Biotec B.V.&Co. KG, Bergisch Gladbach, Germany). Eryptosis results were given as percentage of phosphatidylserine-positive (eryptotic) RBCs. RBCs from blood donors were used as a negative control, and positive controls were obtained by incubating RBCs with ionomycine, as described elsewhere (38).

This study was approved by the institutional review board (IRB) of Charité - Universitätsmedizin Berlin (EA2/092/25).

## Results

To specifically investigate the unexpected effects observed in patient No. 1 following the administration of two doses of sutimlimab, we conducted serological analyses using samples collected before and after treatment. These analyses aimed to assess hemolysis, eryptosis, and C3d-loading on RBCs.

In addition to the stark elevation of free hemoglobin in the plasma of patient No. 1, as a marker of intravascular hemolysis (**Figures 1**, **2A**), patient’s RBCs exhibited a significant increase in eryptosis after the second dose of sutimlimab (4,1% vs. 1,5% eryptotic RBCs, **Figure 3**).

**Figure 3:**
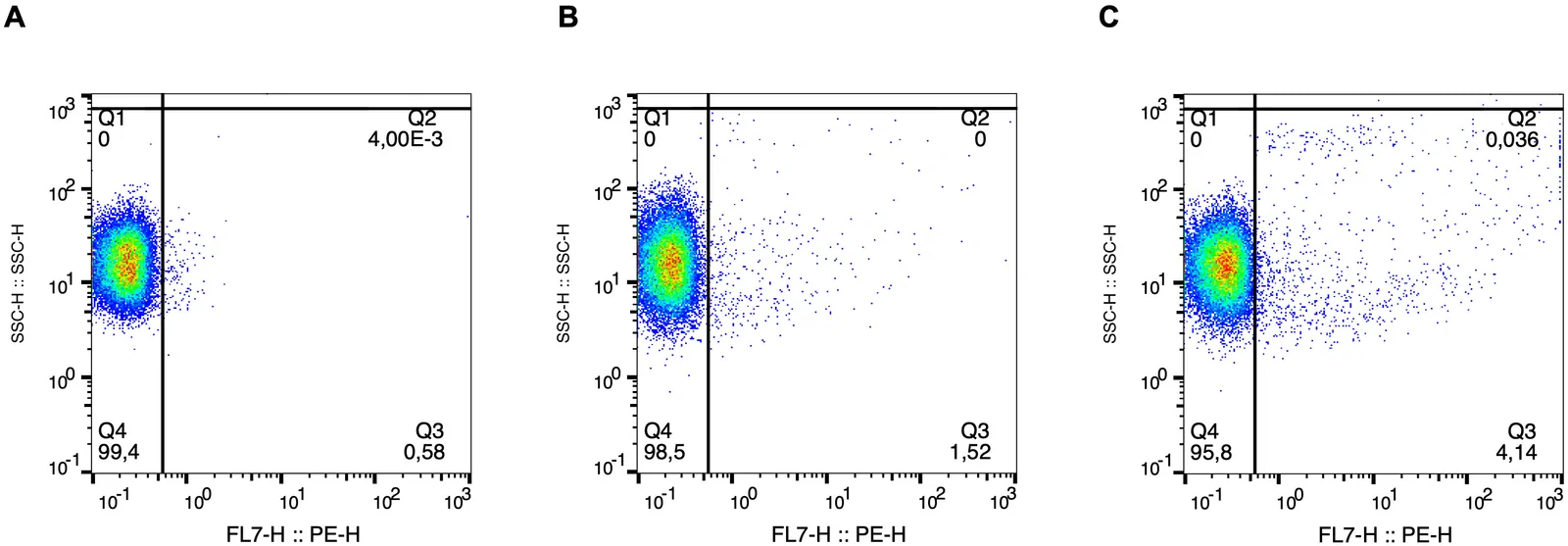
Eryptosis in patient No. 1 before and after sutimlimab treatment. Annexin-PE binding to eryptotic, phosphatidylserine-expressing RBCs. Scatter plot of RBCs from a healthy blood donor **(A)**, patient No.1 before sutimlimab treatment **(B)** and after the second dose of sutimlimab **(C)**.

In the hemolysis assay, the patient’s pre-treatment serum (diluted 1:10 in fresh donor serum) partially induced hemolysis of donor RBCs, as evidenced by elevated levels of free hemoglobin (fHb 0,35 g/dl, **Table 1**). The remaining non-hemolyzed RBCs became strongly coated with C3d. In addition, about 5% of RBCs were eryptotic. In contrast, the patient’s serum collected two days after the second sutimlimab administration did not induce hemolysis (fHb: 0,06 g/dl), or complement activation on RBCs (**Table 1**). Remarkably, RBCs remained strongly agglutinated even after repeated washing at 37 °C, and separation of single cells was only achievable by vigorous pipetting and vortexing. The mechanically dissociated RBCs exhibited a markedly elevated proportion of PS-exposing RBCs (30–50%); however, this is likely primarily attributable to artifactual PS externalization triggered by the shear stress generated during forced mechanical separation, rather than representing true eryptosis. Accordingly, the accurate quantification of eryptosis under these conditions was not feasible. The degree of agglutination was substantially greater than that observed with RBCs treated with the patient’s serum prior to sutimlimab administration, where RBC agglutination was completely reversible after three washing steps at 37 °C.

**Table 1:** Hemolysis and eryptosis of donor RBCs following incubation with serum from patient No. 1 prior to sutimlimab treatment and 2 days after second dose of sutimlimab.

Patient’s serum, diluted 1:10 in fresh donor serum	fHb g/dl	Eryptosis %	C3d-AGT of donor RBCs
prior to treatment	0,35	5	4+
2 days after treatment	0,06	NE	–
prior to treatment + Sutimlimab *in vitro*	0,08	NE	1+
prior to treatment (heat-inactivated)	0,03	NE	–
control serum	0,05	0,5	–

fHb, free hemoglobin in supernatant; C3d-AGT, antiglobulintest with anti-C3d; NE, not evaluable.

Agglutination strength (–): negative; 1+ weak; 2+ moderate; 3+ strong; 4+ very strong.

The absence of hemolysis, absence of C3d deposition on RBCs, and the presence of very strong agglutination were also observed upon the addition of sutimlimab *in vitro* to a mixture of patient’s pre-treatment serum and donor serum (**Table 1**). Comparable results were obtained using heat-inactivated patient’s and donor serum (**Table 1**). These experiments demonstrated that inhibition of complement activation, whether achieved by sutimlimab or by heat inactivation of the patient’s serum, prevented complement-dependent hemolysis, but resulted in increased agglutination.

To further investigate the role of complement on cold aab mediated agglutination, we compared the agglutination properties of the patient’s plasma vs the patient’s serum. The agglutination titer of the patient’s serum and plasma samples (diluted in saline) at room temperature was 256 in both cases (**Table 2**). In contrast, the patient’s plasma diluted in donor plasma yielded a considerably higher titer than the patient’s serum diluted in donor serum (16384 versus 512, **Table 2**). Moreover, donor RBCs showed maximal agglutination (4+) with diluted patient’s plasma up to a dilution of 1:2048 (**Supplementary Table 1**). In contrast, the patient’s serum diluted in fresh donor serum agglutinated donor RBCs only weakly to moderately (**Supplementary Table 1**). A strongly positive C3d antiglobulin test (AGT) of donor RBCs was observed up to a dilution of 1:4096 in the presence of diluted fresh donor serum (**Supplementary Table 1**).

**Table 2:** Cold agglutinin titers at 20 °C using plasma or serum from patient No. 1 prior to treatment.

Donor RBCs	Patient’s sample	Diluent	Titer
untreated	plasma	saline	256
untreated	serum	saline	256
untreated	plasma	donor plasma	16384
untreated	serum	donor serum	512
untreated	serum + sutimlimab	donor serum + sutimlimab	16384
coated with C3d	plasma	donor plasma	256
C3d-coating inhibited by sutimlimab	plasma	donor plasma	16384

Likewise, the addition of sutimlimab to the patient’s serum diluted in donor serum resulted in an increase in the agglutination titer to 16384 (**Table 2**), and the RBCs used in the assay were C3d-AGT negative.

Next, we tested the effect of C3d coating of donor RBCs on agglutination titers of the patient’s plasma diluted in donor plasma (**Table 2**). Agglutination titers obtained with C3d-loaded RBCs were substantially lower than those observed with untreated group O RBCs (titer 256 versus 16384, respectively). When C3d deposition on RBCs was inhibited by sutimlimab, the agglutination titer was comparable to that of untreated cells (titer 16384, **Table 2**), indicating that the pronounced effect of sutimlimab on agglutination is attributable to the prevention of C3d-binding to RBCs.

To extend these findings to a broader patient population and evaluate the generalizability of this effect, we assessed the overall impact of sutimlimab on RBC agglutination by measuring serum agglutination titers at 20 °C, both in the presence and absence of sutimlimab, in a cohort of six patients with clinically significant CAD, comprising the two index patients and four additional patients (**Figure 4**).

**Figure 4:**
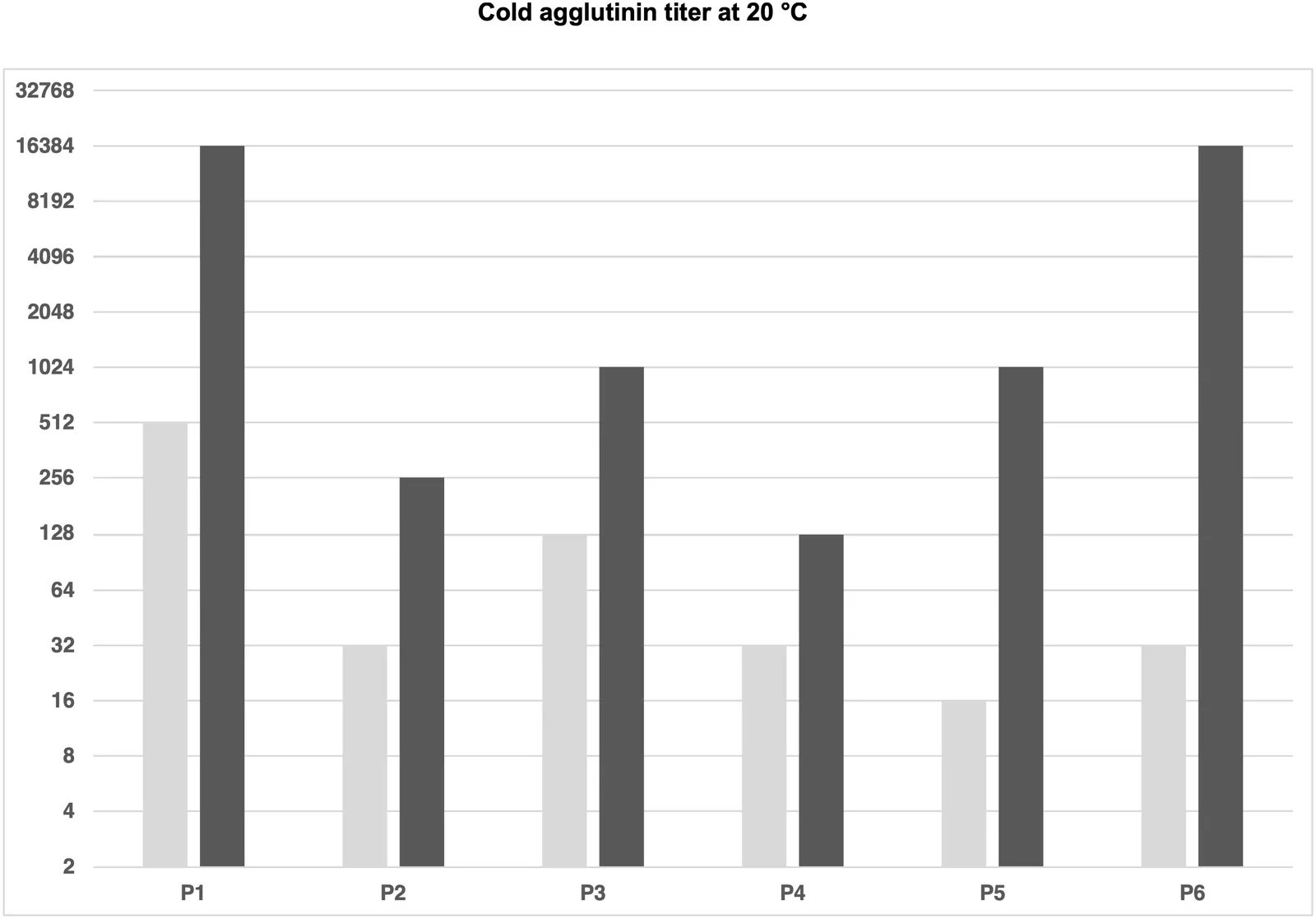
Agglutination titers at 20 °C of patients nos. 1 – 6. First column of each patient: titer of patients’ serum in fresh donor serum; second column: titer of patients’ serum in fresh donor serum, both preincubated with sutimlimab.

In all cases, the addition of sutimlimab resulted in variable increases in RBC agglutination titers. In three patients (Nos. 2, 3, 4), the serum titer increased by only four- or eightfold following the addition of sutimlimab. In contrast, the titer increased between 32- and 512-fold in the remaining patients (Nos. 1, 5, 6), and two patients (Nos. 1 and 6) demonstrated exceptionally high titers (16384).

In conclusion, sutimlimab is highly effective in inhibiting complement-mediated hemolysis, but may infrequently aggravate RBC agglutination in CAD (**Figure 5**).

**Figure 5:**
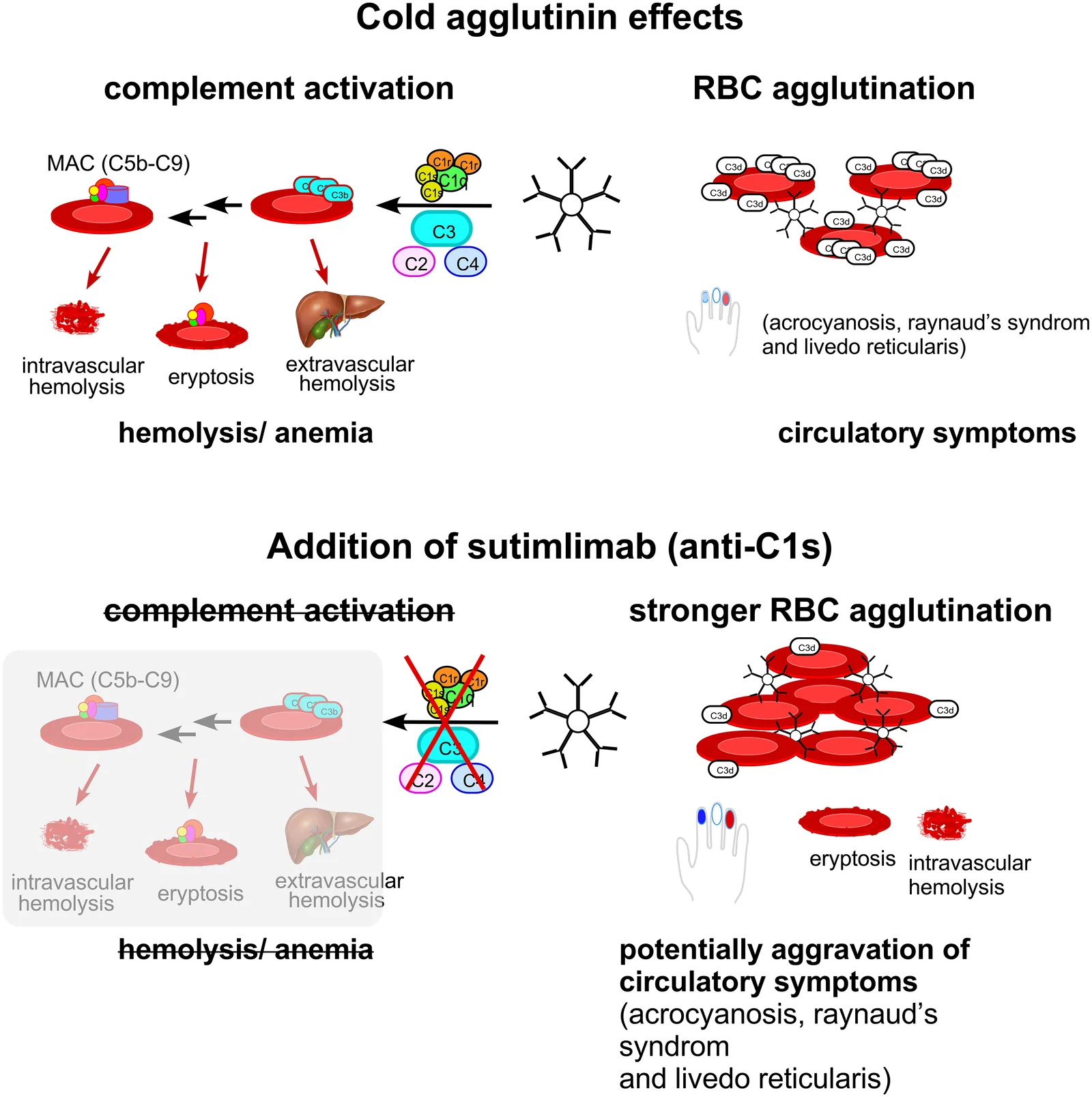
Summary of cold agglutin effects and sutimlimab interactions.

## Discussion

The clinical presentation of CAD is highly variable. The majority of patients experience only mild (Hb 10 g/dl – lower limit of normal) to moderate (Hb 8–10 g/dl) extravascular hemolysis (39) In some cases, patients may develop acrocyanosis, which affects the ears, nose tip, fingers, and toes, typically triggered by exposure to the cold. Symptoms of acrocyanosis generally resolve quickly upon rewarming. Intravascular hemolytic episodes, on the other hand, are rare (1, 5, 12). However, many affected patients require treatment due to significant hemolytic and/or non-hemolytic symptoms (39, 40). In general, numerous factors should be considered before initiating treatment in patients with CAD. These include individual patient characteristics, the severity and types of symptoms, treatment associated risks, concomitant diseases, and the need to balance efficacy with safety. Notably, most available treatments for CAD – whether older or newer therapies – are immunosuppressive.

Ultimately, CAD is an indolent B-cell lymphoproliferative disorder, which may become destabilized during immunosuppressive treatment (41–43). Cold aab with high thermal amplitude can significantly contribute to hemolysis through eryptosis (14). In such cases, the administration of erythropoietin has been shown to improve outcomes (44–46).

Until recently, the most effective treatment for patients with severe cold-induced circulatory symptoms involved immunosuppression with rituximab and bendamustine (39). However, this combination therapy does not provide the rapid efficacy seen with sutimlimab. Sutimlimab, which selectively targets the C1s subunit of the classical complement pathway, has become an increasingly utilized therapy for CAD. Clinical trials have demonstrated its ability to rapidly inhibit hemolysis, and it has generally been well tolerated. However, various side effects - including headache, hypertension, infection, nausea, abdominal pain and acrocyanosis - have been reported (47, 48). Similar side effects have also been observed with other complement inhibitors, such as pegcetacoplan (C3 inhibitor) (24) and iptacopan (complement factor B inhibitor) (25). Importantly, the long-term real-world safety and efficacy of sutimlimab remain incomplete understood.

In this report, we describe two patients who developed alarming side effects during treatment with sutimlimab. Patient No. 1 abruptly developed a hypertensive crisis, requiring hospitalization. This patient also experienced a paradoxical exacerbation of hemolysis, as reflected in laboratory findings showing marked increase in LDH, bilirubin, free hemoglobin and renal insufficiency. These findings represent previously unreported adverse effects of sutimlimab. The mechanism underlying this increased hemolysis remains unclear, but four potential explanations should be considered: 1. enhanced eryptosis resulting from stronger RBC agglutination, 2. increased extravascular phagocytosis, 3. intravascular hemolysis caused by shear stress due to increased RBC agglutination and, less likely, 4. reactive hemolysis mediated by C5-C9 activation independent of C3 (49). Regardless of the mechanism, these findings suggest that sutimlimab may aggravate hemolysis in isolated cases of CAD. In contrast, patient No. 2, experienced a complete remission of hemolysis but developed a wide spread cyanosis that could not be attributed to hemorrhage diathesis or exacerbated acrocyanosis. This patient also reported intermittent episodes of thoracic and abdominal pain, which appeared to be related to sutimlimab therapy. These symptoms may reflect compromised peripheral circulation. Indeed, peripheral circulatory symptoms such as acrocyanosis and Raynaud’s syndrome have been observed in clinical studies of sutimlimab for CAD (32, 33). In the CADENZA trial, 18.2% and 13.6% of sutimlimab treated patients experienced Raynaud’s phenomenon and acrocyanosis, respectively, compared to 0% in the placebo group (29).

A possible explanation for these side effects may lie in increased RBC agglutinability in the absence of complement (C3d) on RBCs in patients treated with sutimlimab. This hypothesis is supported by our *in vitro* experiments, which demonstrate significantly greater cold agglutinin attachment to RBCs in the presence of sutimlimab and absence of complement (C3d) (**Table 2**; **Figure 4**).

In addition, we have previously reported on a lethal AIHA with massive circulatory complications due to RBCs agglutination induced by IgA aab (50). In that study, we demonstrated that strongly hemagglutinating aab may not only cause hemolysis but also hypoxia due to intravascular hemagglutination. Interestingly, progressive splenomegaly and worsening hemolysis has recently been described in a CAD patient under treatment with sutimlimab and pegcetacoplan (51). Whether these hemolytic episodes were a result of RBC destruction due to gross agglutination and eryptosis remains to be answered. Nevertheless, given the limited number of patients in the presented study, our findings should be confirmed in further studies.

Previous studies have shown that cold agglutinin binding is markedly reduced in the presence of C3d-coated RBCs. The deposition of C3d fragments on the RBC membrane, resulting from complement activation, creates a physical barrier that sterically hinders cold agglutinin (IgM antibody) binding and RBC agglutination. This mechanism helps to protect RBCs from further immune-mediated damage. Using ^131^I-labeled cold agglutinins, Evans et al. demonstrated that C3d-coated RBCs in patients with CAD not only inhibit hemolysis, but also decreased further cold agglutinin binding and agglutination through steric hindrance. Notably, normal RBCs from healthy adults adsorbed 2- to 30-fold more cold agglutinins compared to C3d-coated RBCs from CAD patients, highlighting the protective role of C3d deposition (2, 3). These findings are supported by more recent reviews, which emphasize that C3d-coated RBCs are less susceptible to further antibody binding, thereby preventing hemolysis (52, 53).

In this context, the observed worsening hemolysis in Patient No. 1 may be attributed to increased eryptosis and, potentially, intravascular hemolysis resulting from heightened RBC agglutination in the absence of complement. This increased agglutination could lead to shear stress and subsequent RBC membrane damage.

While our findings represent rare adverse events, they may provide valuable insights into the underlying pathology of potential circulatory side-effects associated with complement inhibition by sutimlimab or similar drugs in CAD. The wide range of agglutination titer ratios with or without sutimlimab observed in our experiments (**Figure 4**) may be explained by variability in I-antigen density or differences in cold agglutinin avidity among patients.

Although many patients with CAD achieve partial or complete stabilization with sutimlimab, this therapy requires biweekly administration, and continuous treatment may result in more frequent and severe side effects than currently recognized. Recent studies have suggested that treatment for CAD should be customized based on the presence of hemolytic versus non-hemolytic symptoms (8).

In light of our findings, certain precautions may enhance the safety of sutimlimab therapy. For example, performing a serological agglutination assay using the patient’s serum and a source of complement, both in the presence and absence of sutimlimab, could help identify patients at risk for aggravated agglutination-mediated circulatory symptoms. Additionally, future studies should investigate whether lower doses of sutimlimab might achieve sufficient efficacy while minimizing side effects in patients with highly reactive cold agglutinins. Finally, our observations may have broader implications for the development of new inhibitors targeting early complement components (C1–C3).

## Conclusion

Many factors should be carefully considered before initiating sutimlimab in patients with CAD. While this drug represents a significant advancement in the treatment of CAD due to its rapid inhibition of hemolysis, the potential for aggravated agglutination-mediated side effects must not be overlooked. Our findings underscore the importance of individualized patient assessment and highlight the need for further research to optimize the safety and efficacy of complement inhibitors in CAD.

## Data Availability

The original contributions presented in the study are included in the article/**Supplementary Material**. Further inquiries can be directed to the corresponding author.
