# Supplementary material for: Complement inhibition by sutimlimab in cold agglutinin disease promotes red blood cell agglutination and may lead to circulatory complications, eryptosis and hemolysis
Source: Front Immunol. 2026 Sep 15;17:1906515. doi: 10.3389/fimmu.2026.1906515 (PMC13619883; doi:10.3389/fimmu.2026.1906515)
Supplement: Supplementary file 1 [file DataSheet1.pdf]

Supplemental Table 1:

Reactivity of patient's cold agglutinins at 20 °C with donor RBCs in the presence and in the absence of complement activation.

Dilution 1/x	Patient's plasma diluted in plasma reactivity	Patient's serum diluted in fresh serum reactivity	C3d-AGT of RBCs
1	4+	4+	-
2	4+	1+ *	4+
4	4+	(+) *	4+
8	4+	(+) *	4+
16	4+	1+	4+
32	4+	1+	4+
64	4+	2+	3+
128	4+	2+	3+
256	4+	2+	3+
512	4+	1+	3+
1024	4+	-	3+
2048	4+	-	3+
4096	3+		3+
16384	1+		2+
65536	-		1+

* supernatant hemolytic; C3d-AGT: antiglobulintest with anti-C3d;

Agglutination strength: (-) negative; 1+ weak; 2+ moderate; 3+ strong; 4+ very strong

Supplemental Table 2:

Reactivity of patient's cold agglutinins with C3d-positive or C3d-negative RBCs

**Dilution 1/x**	**Untreated O RBCs**	**O RBCs pretreated with cold agglutinins**	**O RBCs pretreated with cold agglutinins + Sutimlimab**
**C3d-AGT of RBCs**	-	4+	-
1	4+	2+	4+
4	4+	2+	4+
16	4+	2+	4+
64	4+	1+	4+
256	4+	1+	4+
1024	4+	-	4+
4096	4+	-	4+
16384	1+	-	2+
65536	-	-	-

C3d-AGT: antiglobulintest with anti-C3d;

Agglutination strength: (-) negative; 1+ weak; 2+ moderate; 3+ strong; 4+ very strong
